# Evaluation of an Attract-and-Kill Strategy Using Long-Lasting Insecticide Nets for the Management of the Brown Marmorated Stink Bug in Northern Italy

**DOI:** 10.3390/insects15080577

**Published:** 2024-07-29

**Authors:** Antonio Masetti, Agata Morelli, Luca Fagioli, Gianfranco Pradolesi, Riccardo Nicoli, Olmo Scagliarini, Maria Grazia Tommasini, Michele Preti

**Affiliations:** 1 Department of Agricultural and Food Sciences, University of Bologna, 40127 Bologna, Italy; antonio.masetti@unibo.it; 2 Consorzio Agrario di Ravenna Test Facility, 48033 Cotignola, Italy; fagioli@consorzioagrarioravenna.it; 3 Terremerse Società Cooperativa Test Facility, 48012 Bagnacavallo, Italy; gpradolesi@terremerse.it; 4 AgriTes, 40057 Granarolo dell’Emilia, Italy; riccardo.nicoli@agrites.it; 5 Centro Agricoltura e Ambiente “Giorgio Nicoli”, 40014 Crevalcore, Italy; oscagliarini@caa.it; 6 Ri.Nova Società Cooperativa, 47522 Cesena, Italy; mgtommasini@rinova.eu; 7 ASTRA Innovazione e Sviluppo Test Facility, 48018 Faenza, Italy; michele.preti@astrainnovazione.it

**Keywords:** *Halyomoprha halys*, integrated pest management, aggregation pheromones, long-lasting insecticide-treated nets, mass trapping

## Abstract

**Simple Summary:**

The brown marmorated stink bug (BMSB) is causing extensive losses in agricultural products, especially tree fruit crops. Attract-and-kill (AK) strategies, which drive pests out of the cash crop to a circumscribed area where control interventions are applied, could be a more sustainable method for suppressing BMSBs while reducing the use of pesticides. This study assessed the effectiveness of an AK strategy against the BMSBs on pear, comparing sites with and without AK stations, consisting of pheromone lures coupled with insecticide-treated nets. The BMSB abundance was monitored using monitoring traps, and the fruit damage was recorded at harvest. In spring and early summer, the AK stations did not decrease pest density nor the fruit damage. Instead, after harvest, fewer BMSBs were detected in the AK sites than in sites without AK stations. Whilst this study supports the efficacy of the lures, the killing method needs to be refined and improved.

**Abstract:**

The brown marmorated stink bug (BMSB), *Halyomorpha halys* (Stål) (Hemiptera: Pentatomidae), is causing extensive economic losses in tree fruit crops. Including attract-and-kill (AK) strategies targeting BMSBs in an integrated pest management framework could reduce the amounts of insecticides sprayed and benefit growers, consumers and the environment. This study evaluated the effectiveness of an area-wide AK strategy across an intensive fruticulture region of Northern Italy, comparing four paired pear sites with and without two AK stations ha^−1^. These stations consisted of long-lasting insecticide-treated nets containing alpha-cypermethrin, baited with the BMSB aggregation pheromone and synergist. BMSB abundance was estimated using black-standing monitoring traps, and fruit damage upon harvest was recorded across all sites. The AK stations did not decrease the BMSB abundance nor the fruit damage, while after harvest significantly lower BMSB captures were detected in the AK sites compared to the control sites. Whilst the lures’ efficacy was corroborated by this research, the killing method requires improvement and refinement.

## 1. Introduction

Invasive species are considered one of the largest threats to agricultural systems [1]. The execution of effective management strategies against invasive species encounters many obstacles due to the precipitous numbers of individuals present, the scarcity or absence of natural enemies and their propensity to outcompete local species [2]. The unprecedented introduction and dispersal of the brown marmorated stink bug (BMSB), *Halyomorpha halys* (Stål) (Hemiptera: Pentatomidae), from Eastern Asia to the USA [3], Europe [4] and South America [5] has ensued devastating losses in agricultural crops, ornamental plants and wild hosts [6,7,8,9]. Crop losses are further exacerbated by the paucity of successful and sustainable management strategies against the BMSB [10]. 

In Italy, the first established populations of the BMSB were detected in the Emilia-Romagna region in 2012 [11]. Since then, the BMSB has prompted major concerns regarding the quality and yields of several agricultural crops, as the climatic conditions across the country allow bivoltine lifecycles, causing multiple pest outbreaks annually [12,13]. In invaded areas, strategies for controlling the BMSB have strongly relied on the use of broad-spectrum insecticides. However, while partly successful in the short term, this strategy is neither economically nor environmentally sustainable and in the long term interrupts the establishment of integrated pest management (IPM) schemes. Furthermore, commercial insecticides for BMSB control may result in an initial knockdown, but the insect’s ability to recover often renders the insecticide treatments ineffective [14,15]. The non-selective nature of these insecticides also impairs the conservation of natural enemies and other beneficial organisms, including non-target arthropods [15,16]. The stringent regulations on pesticide use in Europe, coupled with the demand to develop control methods that adhere to IPM principles, have driven research toward alternative and more sustainable control techniques. 

Currently, control methods such as exclusion nets [17,18,19] and biological control programs [20,21,22] are being examined but are still at a preliminary stage and require elaboration. The availability of the BMSB two-component aggregation pheromone [23] and pheromone synergist [24] has led research into developing management strategies centered around behavioral manipulation that could prevent or lessen crop losses [25,26]. These pheromones have been refined and optimized [27] and are commercially available and globally used in BMSB monitoring programs [28,29,30]. The pairing of pheromones with an appropriate killing agent could be conducive to developing an attract-and-kill (AK) strategy. In contrast to full-block sprays with broad-spectrum insecticides, AK techniques minimize the contact between pesticides and the crop, as the application is restricted to a precise area whilst potentially maintaining a level of pest control that is comparable to that of standard grower methods. The behavioral basis of the BMSB lends itself to be exploited by an AK strategy. For instance, the BMSB is a perimeter-driven pest, with most damage occurring at crop and fruit orchard edges [31,32]. It is also a landscape-level threat, preferentially colonizing wild or cultivated hosts depending on its nutritional requirements and the crop phenology at that given moment [7,14]. This landscape-level threat is further aggravated when considering the BMSB dispersal capacity [33]. Accordingly, AK stations would be best suited at the perimeter of orchards to intercept the mobile adults and nymphs [34] and should be deployed with an area-wide approach.

Much literature exists on the application of AK strategies for a variety of pests (see, for example, [35,36,37]), with its success varying depending on the pest targeted and the crops. AK has been attempted for other stinkbug management [38], but for the BMSB, it has only been tested in apple orchards in the Mid-Atlantic region of the USA [34,39]. Morrison III et al. [39] reported the first successful account of AK against the BMSB and found an acceptable reduction in crop damage in apple orchards in the United States. However, this technique is not compliant with EU regulations and would need to be adjusted if applied in a European context. For instance, long-lasting insecticide-treated nets (LLINs) could be a practical killing method and have been helpful resources for the control of various lepidopteran and hemipteran pests of vegetable crops [40,41,42]. Some bioassays [43] and small-scale pilot studies [44] testing the efficacy of LLINs on the BMSB have been carried out in Italy, attesting to their potential integration in AK strategies. Nonetheless, this is the first study to explore AK using pheromone baits and LLINs, deployed as an area-wide approach to controlling the BMSB within a European context.

This study was aimed at testing the efficacy of an AK strategy for BMSB management and was conducted in the Po Valley farmlands of the Emilia-Romagna region due to the intensive pest pressure exerted by BMSB. The specific objectives of this research were to (1) compare the population densities of BMSB in sites in which AK was applied to analogous control sites without AK, (2) compare the level of damage to the fruits observed between the orchards with and without the application of AK and (3) critically discuss the technical issues, feasibility and side-effects of developing and promoting AK strategies, specifically in a European context.

## 2. Materials and Methods

### 2.1. Experimental Design and Locations 

**Figure 1:**
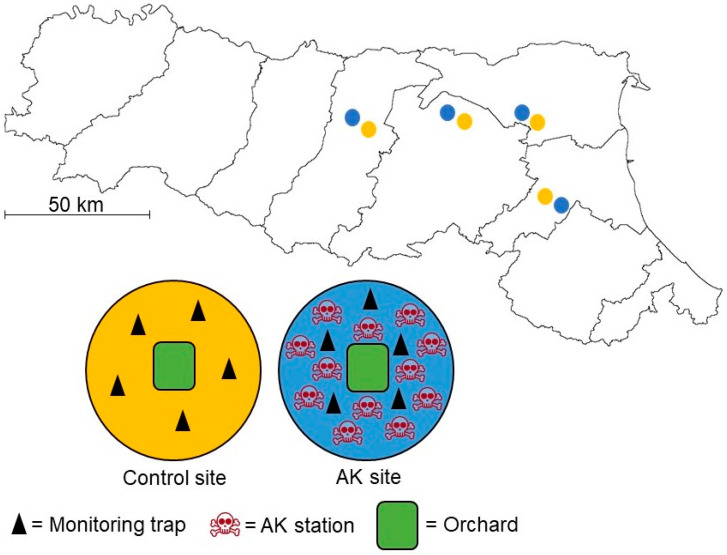
Locations of the sampling sites in Emilia-Romagna (Northern Italy), where orange and blue circles represent the control and AK strategies, respectively, and the arrangement of the AK and monitoring stations in relation to the experimental sites. The partitioning within the Emilia-Romagna region represents the provinces. Exact geographic positions can be found in Table S3.

This study was conducted using an area-wide approach from the beginning of April to the end of October 2021 in Emilia-Romagna, a region in Northern Italy with a high fruit-orchard vocation. A complete randomized block design with two treatments and four replicates was deployed. In each block, two sites ranging between 5 and 20 ha were identified and randomly assigned to either an “AK” or “control” treatment. The minimum distance between sites of the same block was 2 km (Figure 1, Table S3). All sites included at least one commercial pear orchard (>1 ha) and were managed with similar IPM practices, complying with the guidelines and regulations of the Emilia-Romagna region. For BMSB management, growers proceeded with their standard insecticide programs in both AK and control sites.

### 2.2. AK Stations

In each block assigned to the AK treatment, two AK stations/ha were set up, whereas no stations were installed in the control sites. The AK stations comprised TRINET^®^ (BASF Italia S.p.A, Cesano Maderno, MB, Italy) long-lasting insecticide-treated nets (LLINs) containing 1.57% alpha-cypermethrin, baited with the PHEROCON^®^ BMSB high-load dual lure (Trècè Inc., Adair, OK, USA), which is an enhanced BMSB aggregation pheromone with fourfold the rate of the standard PHEROCON^®^ BMSB monitoring dual lure. These lures were baited with the two-component aggregation pheromone, (3*S*,6*S*,7*R*,10*S*)-10,11-epoxy-1-bisabolen-3-ol and (3*R*,6*S*,7*R*,10*S*)-10,11-epoxy-1-bisabolen-3-ol, and the pheromone synergist, methyl (2*E*,4*E*,6*Z*)-decatrienoate. The LLINs were mounted on tripods to form pyramids, which, combined with the aggregation pheromone lure, created the AK stations. The aggregation pheromone lures were replaced every 12 weeks throughout the experimental period, following the directions provided by the manufacturer. 

The AK stations were placed on the outer perimeters of the crops to not hinder farm practices covering as evenly as possible the whole area of the sites. No stations were placed inside the orchards and within 10 m from the orchard borders to avoid the possible aggravation of fruit damage due to the aggregation of BMSB individuals in the surroundings of the AK stations. Some AK stations were also placed near the farm buildings from where the overwintered BMSB adults could start their migration to the orchards in early spring [45]. 

### 2.3. Monitoring Traps

To estimate the relative BMSB density, black-standing monitoring traps (Dead-Inn Pyramid Trap^®^, AgBio Inc., Westminster, CO, USA) were placed in both sites of each block. The monitoring traps were made of heavy-duty corrugated plastic (≈120 cm tall and ≈50 cm wide at the base) and were baited with standard PHEROCON^®^ BMSB monitoring dual lures (Trécé Inc., Adair, OK, USA), which were replaced every 12 weeks, respecting the guidelines indicated by the manufacturer. Five monitoring traps were deployed both in control and AK sites in uncultivated areas and at least 10 m away from orchards (Figure 1). In AK sites, at least 50 m between AK stations and monitoring traps was always kept. 

### 2.4. Insect Sampling and Damage Evaluation

The monitoring traps were served weekly throughout the sampling season to count the BMSB individuals and to remove all the insects caught. Adults and nymphs of BMSBs were separately recorded but then pooled together for all the statistical analyses.

A visual survey of fruit damage at harvest was conducted by selecting 10 fruits from 20 randomly selected pear trees within the middle rows of each orchard. Only Conference and Abate Fetel cultivars, which were grown in three out of four sites, were considered. Given that stink bugs other than BMSBs and mirids have never been a significant concern for pears in Emilia-Romagna, any deformations or dimples on fruits were considered as damage due to BMSB trophic activity.

The injured fruits were ranked into four arbitrary classes of damage due to BMSBs according to the severity of the symptoms conveyed.

- Class 0 = No damage: absence of any injuries;
- Class 1 = Slight damage: fruit surface with one or two deformations and/or dimples;
- Class 2 = Moderate damage: fruit surface with three to five deformations and/or dimples;
- Class 3 = Severe damage: fruit surface with > six deformations and/or dimples.

### 2.5. Data Analysis 

Generalized linear mixed models (GLMM) were used to test the effects of treatments (AK vs. control) on mean monthly captures by the black monitoring traps pooling BMSB nymphs and adults and all the traps in each site (Tables S1 and S2). Sampling months were included as repeated measures, and blocks were included as random factors (treatments were nested within blocks). The interaction treatment × month was also tested, and the Kenward–Roger method was used to estimate degrees of freedom. Because of the huge differences between the early and the late periods of the sampling seasons, two separate models were run to analyze captures before the fruit harvest (i.e., from April to July) and after the pear harvest (i.e., from August to October). Based on comparisons of the Akaike information criterion among models, a gamma error distribution with a log link function and a scaled identity covariance structure between repeated measures were selected in both parts of the season. 

An index of damage caused by BMSBs on pears was calculated using the Townsend–Heuberger formula [46]:

(1) $$damage\;index\left(\%\right)=\frac{\sum vN_{v}\times v}{\left(n-1\right)\times N_{t}}\times 100$$

where *n* is the number of the classes of damage; N*_v_* is the number of fruits in each class of damage; *v* is the value of the different classes of damage (from 0 to 3) and N_t_ represents the total number of pears sampled.

An ordinal logistic regression with a probit link function was used to analyze the effects of AK stations and pear cultivars (Abate Fetel vs. Conference) on ranked data of fruit damage. Analyses were carried out with IBM SPSS Statistics (ver. 26).

## 3. Results

### 3.1. Estimation of BMSB Density

**Figure 2:**
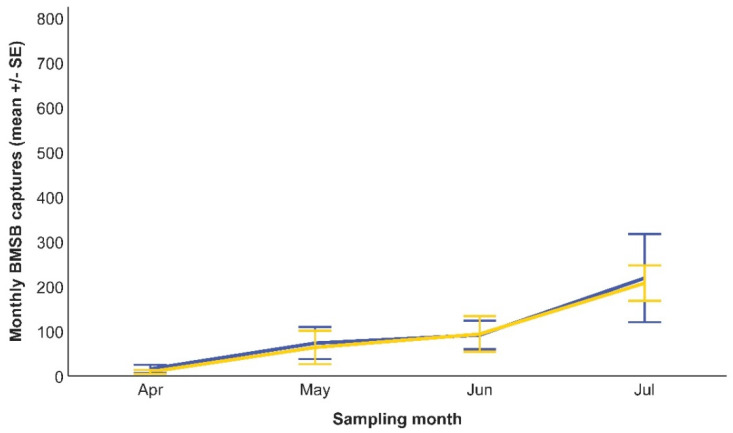
*Halyomorpha halys* individuals caught by black-standing monitoring traps deployed either in AK sites (blue line) or in controls (yellow line) in the early part of the season (April–July 2021). The GLMM did not detect any significant effects on the captures due to the presence of AK stations.

**Figure 3:**
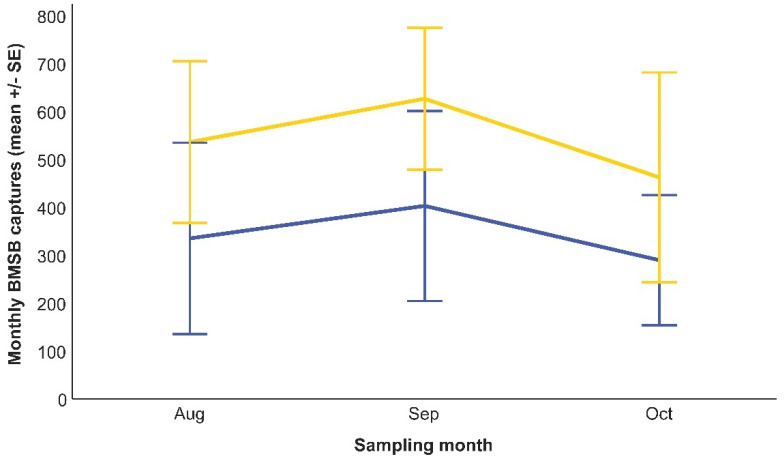
*Halyomorpha halys* individuals caught by black-standing monitoring traps deployed either in AK sites (blue line) or in controls (yellow line) in the late part of the season (August–October 2021). A significant reduction in captures was detected by the GLMM in the sites where AK stations were set up compared to the control sites.

Overall, 13,691 BMSB individuals were caught by the black-standing monitoring traps across the sampling season, of which 5702 individuals pertained to sites where AK stations were established, and 7989 individuals were caught in sites without AK stations. Both BMSB adults (8858 individuals; 64.7%) and nymphs (4833 individuals; 35.3%) were caught. The monthly trend of captures was different between sampling periods. In the early part of the season, before fruit harvest, the number of BMSB individuals caught was low (Figure 2), and no differences between the AK sites and controls were detected (Table 1). On the other hand, the GLMM detected a significant impact of AK stations on the BMSB population density after fruit harvest (Table 2), with a lower monthly mean of insects inside the black-standing monitoring traps deployed in AK sites than in controls (Figure 3).

### 3.2. Fruit Damage

The mean (± standard deviation) damage indexes calculated for Abate Fetel pears were 18.1 ± 3.4% and 9.7 ± 6.1% for the AK and control, respectively. On cv Conference, a mean damage index of 11.1 ± 0.7% was recorded in the AK sites, whereas the mean damage index in the control sites was 4.2 ± 2.1%.

The ordinal logistic model with the probit link function fitted the ranked data of fruit damage (−2 Log Likelihood = 64.8, χ^2^ = 148.8, df = 2, *p* < 0.001; Pearson goodness of fit χ^2^ = 8.6, df = 7, *p* = 0.29; Deviance goodness of fit χ^2^ = 9.5, df = 7, *p* = 0.22). However, the pseudo r square values (Nagelkerke = 0.08; McFadden = 0.04) indicated a small amount of explained variance in the rank of fruit damage due to the presence of AK stations and the pear cultivar. For both predictor variables, significant effects on fruit damage were detected (Table 3). Pears sampled in AK sites had a 1.82 times higher likelihood of falling in the higher categories of damage. Furthermore, the Abate Fetel pears had a tendency (1.54 times) to fall in higher damage classes than the Conference pears.

## 4. Discussion

Since the introduction and widespread invasion of the BMSB in agricultural systems outside its native Asian range, management strategies have almost exclusively relied on broad-spectrum insecticides. AK strategies offer promising contributions in an IPM framework, including the potential to reduce pesticide use, optimizing precision agriculture by confining the problematic areas where pest management interventions are required and safeguarding natural enemies [47]. However, the experimental implementation of an AK method in Northern Italy did not achieve satisfactory results. This study was conducted over a single growing season, and this limited temporal extent acts as a drawback in this research. Nonetheless, the conditions of the studied sites are common in several fruit-growing areas in Southern Europe, and thus, the results could be transferable to areas wider than the Emilia-Romagna region. 

Before harvest, no significant decline in BMSB density by the AK intervention was found. This trend then translated into no cutback in fruit damage at harvest in sites where AK was deployed. Conversely, during the late season, AK stations significantly reduced the pest density compared to the plots where only standard grower methods were practised. Bivoltine lifecycles have been reported for BMSBs in Northern Italy [12], and this increases its threat to agricultural systems, especially in the late season [32,48]. Therefore, successfully reducing pest density in the late season in AK sites may alleviate some of the pest pressure exerted by the second generation. 

However, suppressing second-generation BMSBs may not be helpful for fruits like pears or peaches, which are harvested before August and likely suffer most of the damage by the overwintered adults and first BMSB generation. In contrast, fruits such as apples or kiwifruit may benefit from the potentially lightened pest pressure of the second generation. This, however, would require further research in apple-growing areas and, eventually, an attempt to install the AK stations only later in the season. Similarly, the possible benefits that could manifest in the following growing year are also worth considering. As BMSB density was lower in AK sites during the late season; the smaller numbers of overwintering adults could lead to a reduced population density and, consequently, fruit damage in the next year. 

Since the discovery of the BMSB two-part aggregation pheromone and synergist, extensive research has validated the efficacy of pheromone lures as effective attractants of the BMSB [49], a consensus that our study further supports. However, the killing methods need to be reassessed and improved for the AK strategy to be considered feasible. The approach previously used by Morrison III et al. [39], consisting of weekly spraying of pesticides on apple trees baited with a pheromone, is curtailed by stringent European pesticide regulations and so could not be replicated in this study. While the authors documented the first successful account of AK against the BMSB in the USA, they also elucidated the high implementation costs and concluded that it was economically unsustainable. 

Additionally, a limitation identified in this research pertains to the LLINs employed. Despite LLINs being advocated as a plausible, cost-effective means to protect crops and as potentially compatible with various AK programs within the EU for pest management, they are constrained by the considerably long amount of time that BMSB adults require to absorb a lethal dose of insecticide [34], and the recovery of knocked-down BMSB individuals is often described [50,51,52]. These trends were mirrored by Sabbatini Peverieri et al. [43], who noted that while alpha-cypermethrin LLINs induced sublethal effects and/or mortality, several BMSB adults recovered from initial intoxication.

Finally, the EU recently banned alpha-cypermethrin, the active ingredient incorporated in the LLINs evaluated in this research. To be integrated into AK programs across Europe, insecticide-treated nets must be equipped with alternative active ingredients approved by current legislation—for instance, using deltamethrin-incorporated nets [41,53,54], which are also commercially available. In fact, Kuhar et al. [41] reported adequate BMSB mortality using deltamethrin-incorporated nets, with heightened efficacy observed as the stinkbugs were subjected to the nets for longer periods. As an alternative to LLINs, mass trapping techniques such as drowning through water-basin traps [55,56], funnel traps, glued screens [57,58] or bimodal or multimodal traps using vibrational or light cues [59,60,61] could serve as a viable means of control, albeit necessitating further development and refinement for the optimal management of the BMSB. These strategies offer promising avenues to address the limitations of the current approach. 

## 5. Conclusions

The rigorous regulations on pesticide use in Europe, coupled with the demand to develop control methods that adhere to IPM principles, have opened the door to research alternative control techniques against the BMSB. Testing an AK strategy against the BMSB within an Italian agroecological system was a pertinent attempt, especially when considering the present crop and fruit losses induced by the BMSB in Northern Italy. Although our findings indicated no reduction in fruit damage with the application of AK, it was still important to assess. Overall, this research supports the consensus in literature that validates the efficacy of the ‘attract’ component of AK, but our results highlight that the ‘kill’ component adopted requires a lot of improvement. Further research is required to develop trapping systems that afford a long enough retention time for BMSBs to absorb a lethal dose or to combine the effective pheromone lures with other management strategies, like mass trapping techniques or the use of trap crops. 

## Figures and Tables

**Table 1:** Output of the GLMM with Gamma error distribution and log link function testing the fixed effects of the treatment (AK vs. Control) and sampling month on the BMSB captures by monitoring traps in the early part of the season (April–July 2021).

	F	df_1_	df_2_	*p*
**Treatment**	0.02	1	21.00	0.91
**Month**	25.72	3	21.00	<0.001
**Treatment × Month**	0.37	3	21.00	0.75

**Table 2:** Output of the GLMM with Gamma error distribution and log link function testing the fixed effects of the treatment (AK vs. Control) and sampling month on the BMSB captures by monitoring traps in the late part of the season (August–October 2021).

	F	df_1_	df_2_	*p*
**Treatment**	20.94	1	15.00	<0.001
**Month**	3.38	2	15.00	0.06
**Treatment × Month**	0.26	2	15.00	0.77

**Table 3:** Parameter Estimates of the ordinal logistic regression with Probit link function carried out to model the ranked fruit damage as a function of treatment (AK vs. Control) and pear cultivars.

	Estimate	SE	Wald	df	*p*	95% CI
**Cultivar = Abate Fetel**	0.43	0.06	53.34	1	<0.001	0.31–0.54
**Cultivar = Conference**	0	.	.	0	.	.
**Treatment = AK**	0.60	0.06	105.03	1	<0.001	0.48–0.71
**Treatment = Control**	0	.	.	0	.	.

## Data Availability

The original contributions presented in the study are included in the article/supplementary material; further inquiries can be directed to the corresponding author.

## Supplementary Materials

The following supporting information can be downloaded at: https://www.mdpi.com/article/10.3390/insects15080577/s1, Table S1: Raw data for the mean monthly BMSB captures by black standing pyramid traps, considering both nymphs and adults, per site during the early 2021 season; Table S2: Raw data for the mean monthly BMSB captured by black standing pyramid traps, considering both nymphs and adults, per site during the late 2021 season; Table S3: The coordinates of the geographic positions of the experimental sites.
