# Supplementary material for: Evaluation of an Attract-and-Kill Strategy Using Long-Lasting Insecticide Nets for the Management of the Brown Marmorated Stink Bug in Northern Italy
Source: Insects. 2024 Jul 29;15(8):577. doi: 10.3390/insects15080577 (PMC11354604; doi:10.3390/insects15080577)
Supplement: Supplementary file 1 [file insects-15-00577-s001.zip › insects-3049945-supplementary.pdf]

Table S1. Raw data for the mean monthly BMSB captures by black standing pyramid traps, considering both nymphs and adults, per site during the early 2021 season.

Treatment	Block	Month	Mean BMSB per day
AK	BO	Apr	0.21
Control	BO	Apr	0.02
AK	FE	Apr	0.01
Control	FE	Apr	0.05
AK	MO	Apr	0.37
Control	MO	Apr	0.67
AK	RA	Apr	0.91
Control	RA	Apr	0.30
AK	BO	May	0.61
Control	BO	May	0.25
AK	FE	May	0.24
Control	FE	May	0.48
AK	MO	May	5.04
Control	MO	May	7.08
AK	RA	May	5.25
Control	RA	May	2.25
AK	BO	Jun	4.68
Control	BO	Jun	0.82
AK	FE	Jun	0.49
Control	FE	Jun	5.86
AK	MO	Jun	5.17
Control	MO	Jun	4.33
AK	RA	Jun	5.17
Control	RA	Jun	3.10
AK	BO	Jul	5.71
Control	BO	Jul	3.83
AK	FE	Jul	0.96
Control	FE	Jul	8.25
AK	MO	Jul	10.27
Control	MO	Jul	10.33
AK	RA	Jul	13.67
Control	RA	Jul	8.56

Table S2. Raw data for the mean monthly BMSB captures by black standing pyramid traps, considering both nymphs and adults, per site during the late 2021 season.

Treatment	Block	Month	Mean BMSB per day
AK	BO	Aug	5.86
Control	BO	Aug	18.79
AK	FE	Aug	2.36
Control	FE	Aug	13.50
AK	MO	Aug	5.46
Control	MO	Aug	6.31
AK	RA	Aug	31.00
Control	RA	Aug	32.88
AK	BO	Sep	9.86
Control	BO	Sep	27.82
AK	FE	Sep	3.81
Control	FE	Sep	16.34
AK	MO	Sep	7.12
Control	MO	Sep	9.18
AK	RA	Sep	32.92
Control	RA	Sep	30.19
AK	BO	Oct	9.79
Control	BO	Oct	12.68
AK	FE	Oct	3.33
Control	FE	Oct	6.80
AK	MO	Oct	3.08
Control	MO	Oct	5.36
AK	RA	Oct	22.40
Control	RA	Oct	36.80

Table S3. The coordinates of the geographic positions of the experimental sites.

Province	A&K site GPS coordinates	Control site GPS coordinates
Bologna	44°41'45.41"N - 11°13'50.73"E	44°45'31.40"N - 11°11'58.40"E
Ferrara	44°44'25.20"N - 11°31'26.10"E	44°42'44.11"N - 11°32'51.50"E
Modena	44°40'41.77"N - 10°51'42.05"E	44°40'27.26"N - 10°52'9.73"E
Ravenna	44°18'17.39"N - 11°59'9.90"E	44°18'3.89"N - 11°58'48.38"E
